# Molecular Dissection of Neurodevelopmental Disorder-Causing Mutations in CYFIP2

**DOI:** 10.3390/cells9061355

**Published:** 2020-05-29

**Authors:** Matthias Schaks, Michael Reinke, Walter Witke, Klemens Rottner

**Affiliations:** 1 Division of Molecular Cell Biology, Zoological Institute, Technische Universität Braunschweig, 38106 Braunschweig, Germany; m.schaks@tu-braunschweig.de; 2 Department of Cell Biology, Helmholtz Centre for Infection Research, 38124 Braunschweig, Germany; 3 Institute of Genetics, University of Bonn, 53115 Bonn, Germany; reinke.michael@uni-bonn.de (M.R.); w.witke@uni-bonn.de (W.W.); 4 Braunschweig Integrated Centre of Systems Biology (BRICS), 38106 Braunschweig, Germany

**Keywords:** WAVE regulatory complex, Arp2/3, protrusion, lamellipodium, CRISPR/Cas9

## Abstract

Actin remodeling is frequently regulated by antagonistic activities driving protrusion and contraction downstream of Rac and Rho small GTPases, respectively. WAVE regulatory complex (WRC), which primarily operates downstream of Rac, plays pivotal roles in neuronal morphogenesis. Recently, two independent studies described de novo mutations in the CYFIP2 subunit of WRC, which caused intellectual disability (ID) in humans. Although mutations had been proposed to effect WRC activation, no experimental evidence for this was provided. Here, we made use of CRISPR/Cas9-engineered B16-F1 cell lines that were reconstituted with ID-causing CYFIP variants in different experimental contexts. Almost all CYFIP2-derived mutations (7 out of 8) promoted WRC activation, but to variable extent and with at least two independent mechanisms. The majority of mutations occurs in a conserved WAVE-binding region, required for WRC transinhibition. One mutation is positioned closely adjacent to the Rac-binding A site and appears to ease Rac-mediated WRC activation. As opposed to these gain-of-function mutations, a truncating mutant represented a loss-of-function variant and failed to interact with WRC components. Collectively, our data show that explored CYFIP2 mutations frequently, but not always, coincide with WRC activation and suggest that normal brain development requires a delicate and precisely tuned balance of neuronal WRC activity.

## 1. Introduction

Branched actin filament networks, created by Actin-related protein 2/3 (Arp2/3) complex, play crucial roles in cell motility, neuronal path finding, and morphogenetic events, such as dendrite branching [1,2,3,4,5]. The heteropentameric WAVE (WASP family verprolin homologue) regulatory complex (short WRC) is the major Arp2/3 complex activator in protrusive structures, such as lamellipodia, growth cones, or dendrite branchlets, each mediating distinct, aforementioned processes [3,4,6,7,8]. WRC itself is controlled by a variety of different signaling inputs, but binding to the small GTPase Rac appears to be the most crucial one [2,7,9,10]. While initially proposed to disassemble into two subcomplexes [11], more recent literature strongly suggests that Rac binding to WRC releases the trans-inhibitory interaction of the Arp2/3 complex-activating WCA (WH2, connecting and acidic) domain of WAVE with CYFIP1/2 (cytoplasmic FMR1 interacting protein 1/2, see below), but without WRC subunit dissociation [7,9,10]. WRC is composed of five different components: WAVE2 (or the paralogs WAVE1/WAVE3), CYFIP1—also called Specifically Rac-associated protein-1, Sra-1 (or its paralog CYFIP2), Nck-associated protein 1, Nap1 (or its hematopoietic paralog Hem1), Abl-interacting protein 1, Abi-1 (or Abi-2/3), and HSPC300, also termed BRICK1 [11,12,13,14]. The CYFIP subunit is the regulatory subunit within WRC. CYFIP is on the one hand sequestering WAVE’s WCA domain and on the other hand receiving signaling input from Rac by binding through two distinct GTPase binding sites, called A and D site, for adjacent and distant to the WCA binding site, respectively. Rac binding is commonly agreed to release the WCA domain from transinhibition [10,15]. Due to the extremely high sequence conservation in respective regions, it is assumed that CYFIP1 and CYFIP2 follow a similar biochemical mechanism in transmitting Rac1 binding to WRC activation.

Evidence for a relevance of WRC and thus Arp2/3-dependent actin remodeling in different tissues is continuously growing [3,4,6,8,16,17]. This view is complemented by the discovery of neurodevelopmental disorders, which coincide with genetic aberrations in the *CYFIP1* locus. Deletions involving 15q11–q13, also harboring the *CYFIP1* locus are relatively common. Many of these rearrangements are associated with abnormal phenotypes including seizure, developmental delay and autism, but deletions affecting *CYFIP1* typically cause a worse phenotype, compared to deletions in these regions not involving *CYFIP1* [18]. Much more direct, however, are recent studies showing de novo mutations in the Rac/WAVE regulatory complex (WRC) pathway to be causative for neurodevelopmental disorders and intellectual disabilities. Two studies found mutations in the *NCKAP1* gene, encoding for Nap1, with unknown functions [19,20]. While loss-of-function mutations have been described for the *WASF1* gene [21], encoding the protein WAVE1, another recent study found mutations in the *RAC1* gene and suggested these mutations to either generate dominant negative or constitutively active alleles [22]. Other studies found mutations in *CYFIP2*, again causative for neurodevelopmental disorders, which were accused of causing gain-of-function with respect to WRC activation [23,24], but experimental evidence for this assumption was hitherto missing.

As a model system to systematically analyze the molecular consequences of CYFIP2 mutations normally occurring in neurodevelopmental disorders, we focused on B16-F1 mouse melanoma cells, in which the *CYFIP1* and *CYFIP2* genes were disrupted using CRISPR/Cas9 [7]. CYFIP1/2 removal causes complete failure to form Rac-dependent lamellipodia, which can be readily restored as a readout system for WRC-mediated actin remodeling by ectopic expression of CYFIP1 [7]. These Arp2/3 complex-rich, lamellipodial actin networks constitute the best-characterized, WRC-dependent structures, but they also display high similarity to growth cones [6]. We propose that results obtained with this cell-based, morphological assay can be directly translated into functions of WRC in similar structures, such as a neuronal growth cone or dendrite branchlet common to the nervous system.

## 2. Materials and Methods

### 2.1. Cell Culture

B16-F1 cell line was purchased from American Type Culture Collection, ATCC (CRL-6323, sex:male). B16-F1 derived CYFIP1/2 knockout (KO) cells (clone #3) were as described. B16-F1 cells and derivatives were cultured in Dulbecco′s Modified Eagle′s Medium, DMEM (4.5 g/L glucose; Invitrogen), supplemented with 10% fetal calf serum, FCS (Gibco, Paisley, UK), 2 mM glutamine (Thermo Fisher Scientific, Darmstadt, Germany) and penicillin (50 Units/mL)/streptomycin (50 µg/mL) (Thermo Fisher Scientific, Darmstadt, Germany). B16-F1 cells were routinely transfected in 35 mm dishes (Sarstedt, Nümbrecht, Germany), using 0.5 µg DNA in total and 1 µL JetPrime for controls, and 1 µg DNA in total and 2 µL JetPrime for B16-F1-derived knockout cells. After overnight transfection, cells were plated onto acid-washed, laminin (Sigma-Aldrich, Taufkirchen, Germany)-coated (25 µg/mL) coverslips and allowed to adhere for at least 5 h prior to analysis. For determining protein half–life, cycloheximide (Abcam, Amsterdam, The Netherlands) was added at a concentration 20 µg/mL for the times indicated, and followed by Western Blotting.

### 2.2. DNA Constructs

Vectors enabling fusion of genes of interest to enhanced green fluorescence protein, EGFP, i.e., pEGFP-C2 and -C3 vectors were purchased from Clontech, Inc. (Mountain View, CA, USA). pEGFP-C2-Sra-1 (CYFIP1), and derived mutant constructs (i.e., A site [C179R/R190D], WCA* [L697D/Y704D/L841A/F844A/W845A] and A site+WCA* [C179R/R190D/L697D/Y704D/L841A/F844A/W845A]) were described previously [7] and correspond to the splice variant *CYFIP1a*, sequence AJ567911. Murine CYFIP2 cDNA was isolated following reverse transcription and amplification from murine brain (320E) or kidney (320K) RNA, by amplifying the CDS as two overlapping fragments using the following primers (5′ to 3′): GAA GAG GAT CTG AAT TAC GAT CGA GGC AAG CCG ATG ACC ACC CAC GTC and ACC AGA GCT GGG AGA GG for 5′ end amplification and TCT CCC AGC TCT GGT TCC GAG and CCG CGG TAC CGT CGA CCA GCA GGT GGT GGC CAA TGA CTG G for 3′ end amplification. Both fragments were joined together with the PvuI/SalI digested pEGFP-N1 harboring an additional N-terminal Myc-tag using Gibson Assembly (NEB, Frankfurt, Germany) according to the manufacturers protocol. Subsequently, the cDNA corresponding to GeneBank-ID AF334144.1 was amplified and transferred into pEGFP-C3 vector for direct comparison with EGFP-tagged CYFIP1 wildtype (WT), using SacI and SalI restriction sites upon PCR-amplification with primers GAGAGAGCTCATGACCACCCACGTCACTTTG and GAGAGTCGACCTAGCAGGTGGTGGCCAAT. CYFIP2-related mutations were introduced into the respective positions in CYFIP1 by site-directed mutagenesis, based on sequence alignment (Figure S1), and CYFIP2-R87C engineered accordingly. The identity of all DNA constructs was verified by sequencing.

### 2.3. CRISPR/Cas9-Mediated Genome Editing

B16-F1 cells lacking functional *CYFIP1* and *CYFIP2* genes, as well as reduced expression of Rac GTPases, were generated by treating CYFIP1/2 KO cells (clone #3) with pSpCas9(BB)-2A-Puro (PX459) vectors targeting Rac1, Rac2, and Rac3 genes, as described [25]. Specifically, cells were co-transfected with plasmids targeting ATGCAGGCCATCAAGTGTG (Rac1/2) and ATGCAGGCCATCAAGTGCG (Rac3) genomic regions as described [7]. For obtaining B16-F1 derived cells expressing reduced levels of CYFIP, B16-F1 cells were co-transfected with plasmids targeting GACAGAAATGCATTTGTCAC (CYFIP1) and GACAGGAATGCATTTGTCAC (CYFIP2) genomic regions, as described [7]. After puromycin selection of transfected cells (3 days), cells were extensively diluted and, a few days later, macroscopically visible colonies picked, to obtain single cell-derived clones. Derived cell clones already lacking CYFIP1/2 were screened for low expression of Rac GTPases by Western Blotting.

### 2.4. Western Blotting

Preparation of whole cell lysates was performed essentially as described [7]. Western blotting was carried out using standard techniques. Primary antibodies used were CYFIP1/2 (Sra-1/PIR121 [14], Rac1/3 (23A8, Merck, Darmstadt, Germany), Nap1 [14], WAVE [7], Abi-1 [7], and Glyceraldehyde 3-phosphate dehydrogenase, GAPDH (6C5, Calbiochem, Schwalbach, Germany). Horseradish peroxidase, HRP-conjugated secondary antibodies were purchased from Invitrogen, Rockford, IL, USA. Chemiluminescence signals were obtained upon incubation with ECL™ Prime Western Blotting Detection Reagent (GE Healthcare, Buckinghamshire, UK), and were recorded with ECL Chemocam imager (Intas, Goettingen, Germany).

### 2.5. Immunoprecipitation

For EGFP-immunoprecipitation experiments, B16-F1 cells expressing EGFP or EGFP-tagged variants of CYFIP1 were lysed with lysis buffer (1% Triton X-100, 140 mM KCl, 50 mM Tris/HCl pH 7.4/50 mM NaF, 10 mM Na_4_P_2_O_7_, 2 mM MgCl_2_, and Complete Mini, EDTA-free protease inhibitor (Roche, Mannheim, Germany)). Lysates were cleared and incubated with GFP-Trap agarose beads (ChromoTek, Martinsried, Germany) for 60 min. Subsequently, beads were washed three times with lysis buffer lacking protease inhibitor, mixed with Laemmli buffer, boiled for 5 min, and subjected to Western Blotting.

### 2.6. Fluorescence Microscopy, Phalloidin Stainings, and Quantification

B16-F1-derived cell lines were seeded onto laminin-coated (25 µg/mL), 15 mm-diameter glass coverslips (Hecht Assistent, Sondheim, Germany) and allowed to adhere for at least 5 h. Cells were fixed with pre-warmed, 4% paraformaldehyde (PFA, Sigma-Aldrich, Taufkirchen, Germany) in phosphate-buffered saline (PBS, Gibco, Paisley, UK) for 20 min, and permeabilized with 0.05% Triton-X100 (Sigma-Aldrich, Taufkirchen, Germany) in PBS for 30 s. PFA-fixed cell samples following transfections with plasmids mediating expression of EGFP-tagged proteins were counterstained with ATTO-594™ (ATTO-TEC, Siegen, Germany)-conjugated phalloidin.

### 2.7. Time-Lapse Microscopy

Live cell imaging was done with CYFIP1/2 KO cells (clone #3) transfected with respective EGFP-tagged CYFIP1 variants and migrating on laminin-coated glass (25 µg/mL). Cells were observed in µ-Slide 4 well (Ibidi, Graefelfing, Germany), and maintained in microscopy medium (F12 HAM HEPES-buffered medium, Sigma-Aldrich, Taufkirchen, Germany), including 10% FCS, 2 mM L-glutamine and penicillin (50 Units/mL)/streptomycin (50 µg/mL) (Thermo Fisher Scientific, Darmstadt, Germany). Conventional video microscopy was performed on an inverted microscope (Axiovert 100TV, Zeiss, Goettingen, Germany) equipped with an HXP 120 lamp for epifluorescence illumination, a halogen lamp for phase-contrast imaging, a Coolsnap-HQ2 camera (Photometrics, Tucson, AZ, USA), and electronic shutters driven by MetaMorph software (Molecular Devices, San Jose, CA, USA). Live cell images were obtained using a 100×/1.4 NA Plan apochromatic oil objective at a frame rate of 12/min. Kymographs were generated using MetaMorph software by drawing lines from inside the cell and across the cell edge, and the protrusion velocity determined by measuring the advancement of cell edges over time.

### 2.8. Calculation of Surface Conservation

Amino acid conservation scores of CYFIP1 within WRC (PDB ID: 3P8C) were calculated using the ConSurf webserver (https://consurf.tau.ac.il/) [26]. A total of 186 unique protein sequences ranging from 30 to 95% identity were used to calculate conservation (scored from 1 to 9) and surface conservation color-coded as indicated in the figure.

### 2.9. Statistical Analysis

To assess statistical significance, two-sided, two-sample t-test was applied when data passed normality and equal variance tests, otherwise nonparametric Mann-Whitney-Rank-Sum test was performed. Statistical analyses were performed using Sigma plot 12.0 (Systat Software, Erkrath, Germany). Observed differences between groups were considered to be statistically significant if the error probability (*p*-value) of this assumption was below 5% (*p* < 0.05).

## 3. Results

**Figure 1:**
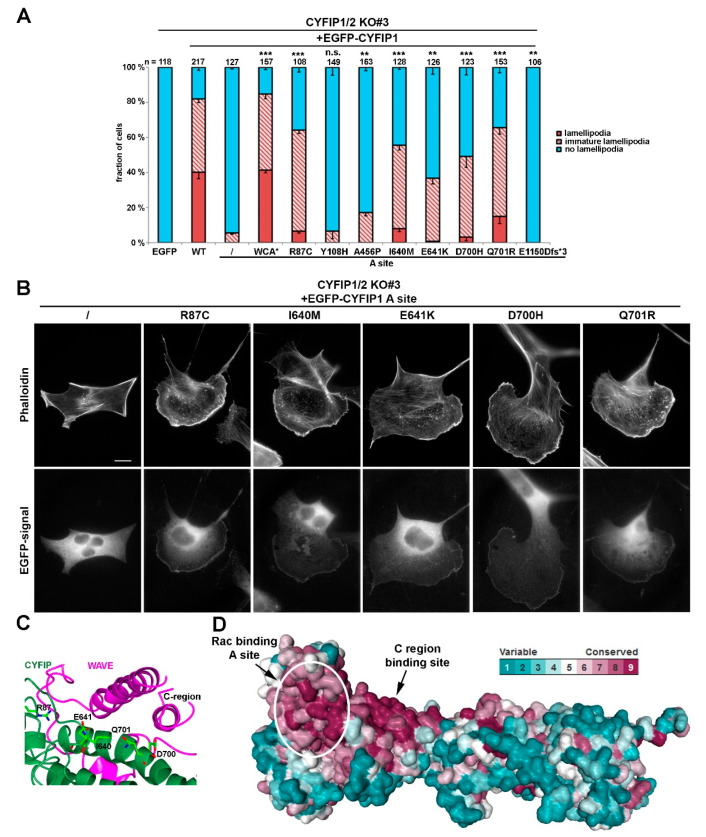
Intellectual disability-causing mutations in CYFIP frequently cause constitutive activation of WAVE regulatory complex (WRC). (**A**) Quantification of lamellipodia formation. Indicated, EGFP-tagged CYFIP1 constructs (or EGFP as control) were transfected into B16-F1 CYFIP1/2 KO cells (clone #3), and lamellipodia formation was assessed after staining of the actin cytoskeleton with ATTO 594-labelled phalloidin. Lamellipodial actin networks that were generally small, narrow, irregular, or displayed multiple ruffles were defined as ‘‘immature lamellipodia’’, as opposed to regular, fully developed lamellipodia [7]. n gives number of cells analyzed, and data correspond to arithmetic means ± SEM from at least three independent experiments. Statistical significance was assessed for differences between the percentages of cells with “no lamellipodia” phenotype. n.s.: not statistically significant; ** *p* < 0.01; *** *p* < 0.001 (two-sample, two-sided t-test). In all cases, statistical analysis was done for the A site mutant alone compared to each individual mutation introduced into the A site-mutated background. (**B**) Cell morphologies of CYFIP1/2 KO cells (clone #3) expressing respective constructs, as indicated. Panels in top row show stainings of the actin cytoskeleton with phalloidin, and bottom row images show fluorescence of the same cells derived from EGFP fluorescence. Scale bar, 10 µm. (**C**) Close up view of the interface between CYFIP and the C-region of WAVE, required for transinhibition of WAVE’s WCA domain. Mutations in CYFIP presumably causing constitutive activation of WRC through abrogation of the transinhibitory WCA-domain interaction are shown. (**D**) Surface conservation of CYFIP. Note that high sequence conservation is found in a larger area of CYFIP contacting the C-region of WAVE, and in which many activating mutations are located.

Our previous results demonstrated that B16-F1 cells disrupted for both CYFIP1 and -2 (Sra-1/PIR121-KO clone #3) lack lamellipodia entirely, but can be restored to form lamellipodia upon expression of CYFIP1 wildtype [7]. It was also established previously that mutational inactivation of one of the two Rac-binding sites in CYFIP1, the so-called A site, abrogated WRC activation, since A site-mutated CYFIP1 was almost entirely abolished in its capability to restore lamellipodia formation (see Reference [7] and Figure 1A). This defect, however, was completely eliminated upon additional mutation of CYFIP1 residues essential for binding to the WCA-domain of WAVE proteins, hence a modification rendering WRC constitutively active, termed WCA* [7,10]. These data unequivocally established that the major role of the A site interacting with Rac is to activate WRC, driving its lamellipodial edge localization and coincident Arp2/3 complex activation [7]. 

To ask whether previously described CYFIP2 mutations found in human patients might indeed also render WRC constitutively active, we transferred these mutations to A site-mutated, murine CYFIP1. Note that murine CYFIP1 and CYFIP2 are 87% identical at the amino acid level and no difference between the two isogenes concerning Rac binding or function within WRC could hitherto be established. An alignment of multiple CYFIP variants from various experimental model systems down in evolution from chicken, fish, and fly until slime mold (*D. discoideum*) and plants revealed, firstly, that all residues mutated in CYFIP2 of human patients are conserved in CYFIP1 of human, mouse, chicken, and fish, and, secondly, that in spite of very few exceptions, the degree of conservation in mutant residues was striking, down to species as distant as *Dictyostelium discoideum* (Figure S1). Notably, our conclusions on the differential regulation of murine CYFIP1 by its two distinct Rac binding sites was fully recapitulated by the same mechanisms in the single *pirA* gene in *Dictyostelium discoideum*, strongly suggesting that respective mechanisms are fundamental and relevant across species barriers [7].

Alongside this notion, we here found that, when re-expressing either CYFIP1 or CYFIP2 in CYFIP1/2 null cells, both were able, in principal, to rescue lamellipodia formation, although CYFIP1 performed moderately better in our cell model (Figure S2A). Thus, and because effects of mutation of the Rac binding sites were already well established in our system for CYFIP1 [7], we initially introduced ID-causing CYFIP2 mutations into the A site-mutated CYFIP1 background. Employing such combinatorial mutations in the CYFIP1/2 double knockout revealed that activation of WRC can also be achieved by mutations alternative to WCA* (Figure 1A,B). While the A site mutant was capable of triggering the formation of at best immature lamellipodia, and only at very poor frequency

(<5% of transfected cells; Figure 1A), additional mutation of CYFIP2 residues found to be modified in patients suffering from ID, specifically R87C, I640M, E641K, D700H, and Q701R (positions in CYFIP1) restored lamellipodia formation to substantial extent, albeit not quite as completely as seen with A site-mutated WCA* (Figure 1A,B). Restoration of lamellipodia formation by these constructs also coincided with their robust accumulation at the tips of lamellipodia now formed, a subcellular localization not seen for the mostly cytosolic, EGFP-tagged A site mutant of CYFIP1 (Figure 1B, left panels). However, since the rescue of lamellipodia formation was not as strong as disrupting the WH2 (W)- and C-region contact sites in CYFIP1 in the WCA* variant, our data indicated a partial rather than full activation of WRC by these patient-derived mutations.

Interestingly, all these residues mutated in patients either directly contact the C-region of WAVE or are positioned at least in close proximity to the C region binding site (Figure 1C). Investigating the evolutionary surface conservation indicates that the C-region binding site of CYFIP is extremely highly conserved (Figure 1D), potentially explaining why modifications in this region result in impaired C-region binding, which is necessary for WCA domain sequestration [10]. Interestingly, the A456P mutation only caused a slight, yet statistically significant increase in lamellipodia formation in these conditions. Moreover, the Y108H mutation did not show any apparent effect in this specific assay, although it was also proposed previously to regulate WRC activation [24].

In our attempts to further characterize the activating potential of specific ID-causing CYFIP mutations, we initially focused on R87C, as this mutant was reported to occur recurrently, in two independent studies [23,24], and caused a robust induction of lamellipodia formation in the context of abrogated A site function (Figure 1A,B). Introduction of the R87C mutation or WCA* into WT CYFIP1 did not change the frequency of lamellipodia formation upon rescue in CYFIP1/2 KO cells (clone #3) (Figure S2B). We conclude this to simply derive from the fact that Rac-mediated WRC activation in B16-F1 cells migrating on laminin is already at an optimum and not, apparently, the limiting factor if restoration of WRC function is undertaken with wildtype CYFIP1 [7]. A potential improvement of activation as seen in the context of the A site mutation can thus not be phenotypically documented in wildtype CYFIP1 conditions.

**Figure 2:**
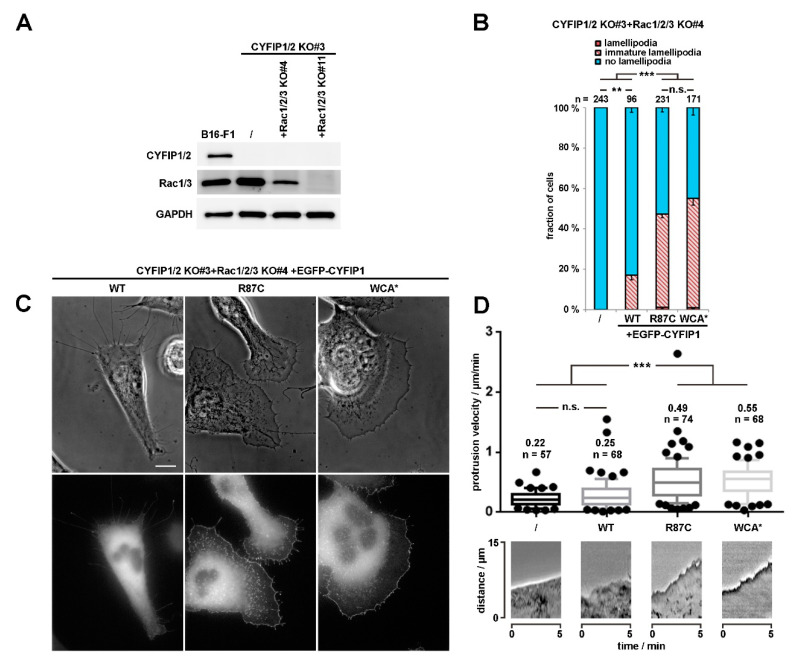
R87C mutation in CYFIP enhances lamellipodium protrusion at low Rac GTPase levels. (**A**) Western blotting of distinct cell lines to probe for expression levels of endogenous CYFIP and/or Rac GTPases. (**B**) Quantification of lamellipodia formation in B16-F1 CYFIP1/2+Rac1/2/3 KO cells (clone #3/4), harboring low Rac expression and transfected with indicated EGFP-tagged CYFIP1 constructs, as described in Figure 1A. Statistical significance was assessed by two-sample, two-sided t-test. n.s.: not statistically significant; ** *p* < 0.01; *** *p* < 0.001 (**C**) Live cell imaging of CYFIP1/2+Rac1/2/3 KO cells (clone #3/4) expressing EGFP-tagged CYFIP1 constructs. Upper panels show phase contrast images, and bottom panels EGFP fluorescence. Scale bar, 10 µm. (**D**) Quantification of cell edge advancement. n gives the number of cells analyzed. Box and whisker plots represent data as follows: boxes correspond to 50% of all data points (25–75%), and whiskers to 80% (10–90%). Lines and numbers above boxes correspond to medians. Statistical significance was assessed by non-parametric, Mann-Whitney-Rank-Sum test, n.s.: not statistically significant; *** *p* < 0.001. Bottom part shows representative kymographs.

**Figure 3:**
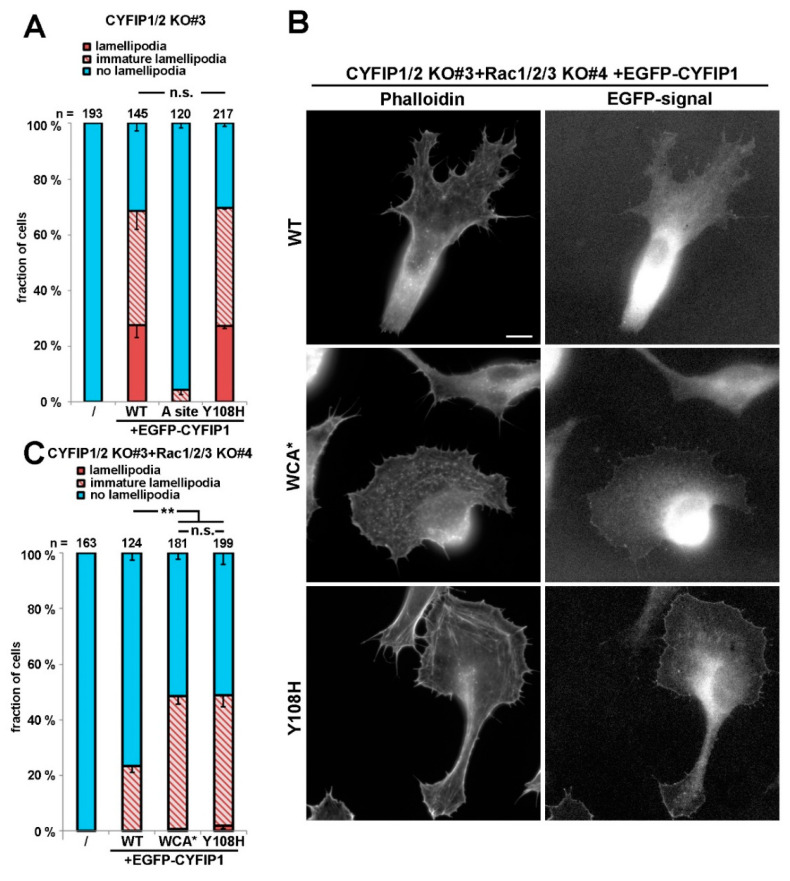
Y108H mutation in CYFIP can augment lamellipodia formation given that the Rac-binding A site is functional. (**A**) Quantification of lamellipodia formation in B16-F1 CYFIP1/2 KO cells (clone #3) transfected with indicated EGFP-tagged CYFIP1 constructs, as described for Figure 1A. (**B**) Representative cell images of CYFIP1/2+Rac1/2/3 KO cells (clone #3/4) expressing respective constructs, as indicated. Panels in left column show stainings of the actin cytoskeleton with phalloidin, and right column images show fluorescence of the same cells derived from respective, EGFP-tagged constructs. Scale bar, 10 µm. (**C**) Quantification of lamellipodia formation in B16-F1 CYFIP1/2+Rac1/2/3 KO cells (clone #3/4), transfected with indicated, EGFP-tagged CYFIP1 constructs, performed in analogy to Figure 1A. Statistical significance was assessed by two-sample, two-sided t-test. n.s.: not statistically significant; ** *p* < 0.01.

However, to illustrate further that the R87C mutation enhances the activation state of WRC *in cellulo*, we also sought to employ a complementary approach, which was independent of introduction of additional mutations in respective CYFIP subunit, as occurring in human patients. To do this, we employed a B16-F1 melanoma clone that had arisen from our attempts to disrupt Rac1/2/3 genes in the CYFIP1/2 null background, as described previously [25]. The novel clone (termed CYFIP1/2 KO#3+Rac1/2/3 KO#4; see Figure 2A), hitherto unpublished, which was also selected upon CRIPSR/Cas9-treatment targeting all Rac alleles, displayed reduced but not entirely abolished Rac expression, as opposed to previously published CYFIP1/2 KO#3+Rac1/2/3 KO#11 (Figure 2A; Reference [25]). We hypothesized that, if reduced Rac expression levels were indeed physiologically relevant for the induction of lamellipodia formation in this new cell line, rescue with WT CYFIP1 would cause a reduced frequency of this response as compared to the parental CYFIP1/2 null clone (see Figure 1), and this was indeed the case. The frequency of CYFIP1/2 KO#3+Rac1/2/3 KO#4 cells capable of lamellipodia formation upon rescue with WT CYFIP1 was observed to be below 20%, but could be significantly increased by both, R87C and again, WCA* mutation (Figure 2B). Moreover, in those WRC-deficient, low Rac expressing cells (CYFIP1/2 KO#3+Rac1/2/3 KO#4), in which lamellipodia were formed upon rescue with R87C or WCA*, these lamellipodia often accumulated WRC at their tips at significantly increased intensities as compared to the ones seen with WT CYFIP1 (Figure 2C). To explore more directly if increased lamellipodia formation frequency (Figure 2B) and WRC accumulation intensity (Figure 2C) was also able to translate into increased actin assembly efficiency, we examined the velocity of protrusion of the cell edges formed in different conditions. We had previously established a clear correlation between WRC gene dose and rate of protrusion or lamellipodial Arp2/3 complex activation (see Reference [27]; for review, also see Reference [2]), so changes in capability to activate WRC should also be reflected in protrusion efficacy. And indeed, whereas WT CYFIP1 on average failed to promote cell edge protrusion beyond the levels observed in B16-F1 lacking endogenous WRC and displaying reduced Rac expression, R87C, as well as WCA*, mutants in these cells led to an increase in protrusion speed by at least two-fold. This strongly suggests that a subset of ID-causing CYFIP2 mutations gives rise to WRCs that can more easily be activated or are already pre-activated, at least partially. To confirm that described mutations can function redundantly in CYFIP2, we introduced the R87C mutation into CYFIP2 as exemplary proof of principle. Although EGFP-tagged CYFIP2-R87C does not promote statistically enhanced lamellipodia formation as compared to EGFP-CYFIP2 in cells displaying wildtype Rac levels (CYFIP1/2 KO#3; Figure S2A, two right hand columns), this is certainly observed when Rac levels are genetically reduced (CYFIP1/2 KO#3+Rac1/2/3 KO#4; see Figure S2C). This confirms that the mechanisms regulating CYFIP1 and CYFIP2 are likely and stringently conserved in the two isogenes. In clear contrast to the majority of patient-derived mutations including R87C, the Y108H mutation did not show altered lamellipodia formation when combined with the A site mutation. However, Y108 is positioned in close proximity to one of the two Rac-binding sites in CYFIP1/2, the so-called A site [10,15] operating in Rac-dependent WRC activation [7], as mentioned above, opening up the possibility that its mutation might influence or modulate Rac binding through this site. Interestingly, no difference was detected in our system when rescuing WRC-deficient cells with wildtype CYFIP1 versus CYFIP1-Y108A (see Figure 3A), indicating at least that the mutation does not impair Rac-mediated WRC activation through the A site. However, in cells lacking WRC and displaying reduced Rac levels, an enhancement of the frequency of lamellipodia formation was observed with the Y108H-mutant, which was virtually identical to the WCA* mutation (Figure 3B,C). This suggests that the Y108H mutation enhances Rac binding to the A site or perhaps eases allosteric activation by this GTPase, or both. In any case, the mutation requires a functional A site to take effect.

**Figure 4:**
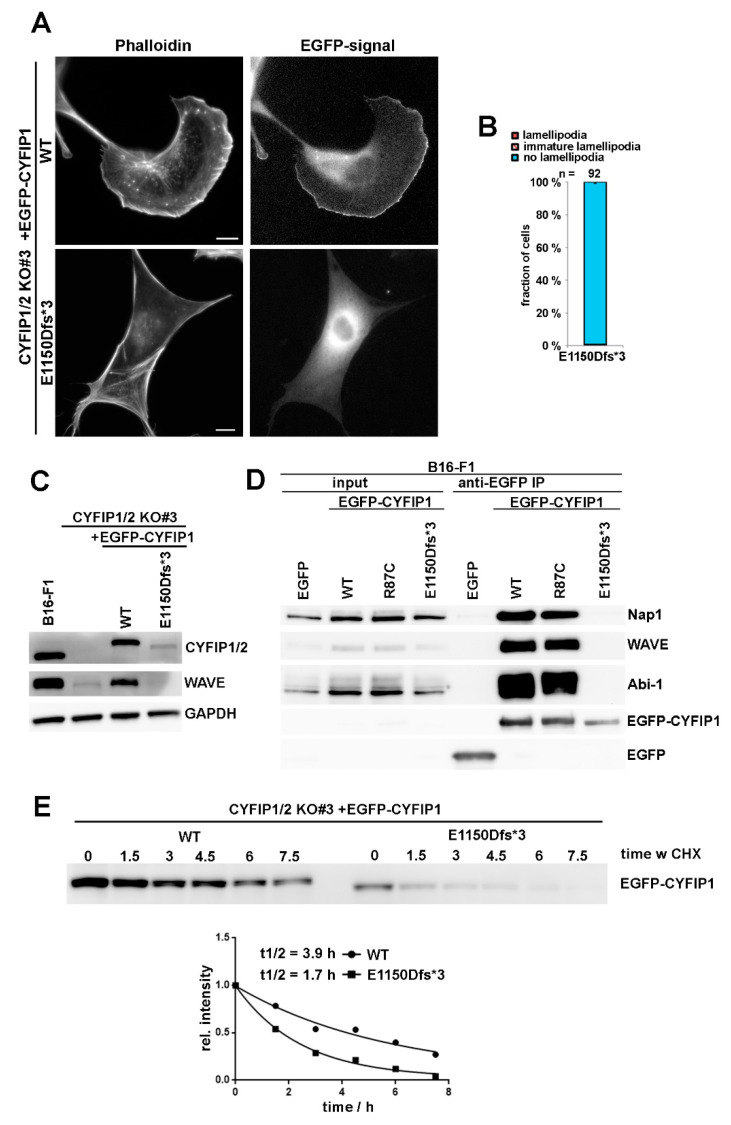
C-terminal truncation of CYFIP prevents WRC assembly and lamellipodia formation. (**A**) Cell morphology of CYFIP1/2 KO cells (clone #3) expressing EGFP-tagged WT or E1150Dfs*3 CYFIP1 mutant, leading to a C-terminally, truncated protein. Scale bar, 10 µm. (**B**) Quantification of lamellipodia formation, done as described for Figure 1A. Note that CYFIP1 WT control is as in Figure 1A. (**C**) Western blotting of B16-F1 cells or CYFIP1/2 KO cells (clone #3) transfected with indicated constructs and probed for expression of CYFIP and WAVE. (**D**) B16-F1 cells were transfected with indicated constructs, lysed and subjected to immunoprecipitation analysis to assay interaction of the E1150Dfs*3 mutant of CYFIP1 versus WT or R87C-CYFIP1 with WRC subunits WAVE, Nap1 and Abi-1. (**E**) Cycloheximide treatment of CYFIP1/2 KO cells (clone #3) transfected with either WT- or E1150Dfs*3-CYFIP1. Cells were treated with 20 µg/mL cycloheximide for indicated times (hours), lysed and cell extracts subjected to western blotting against CYFIP1 (top). The decay of protein expression was plotted over time and derived protein half-life estimated. One representative experiment out of two is shown.

As opposed to R87C and most other patient-derived mutations, the truncating mutation E1150Dfs*3 caused a complete failure in triggering lamellipodia formation in A site-mutated CYFIP1 (Figure 1A), which already indicated that this mutation cannot likely generate a variant that is constitutively activated. To directly test its capability of rescuing lamellipodia formation in CYFIP1/2 null cells, the modification was introduced into the background of WT CYFIP1, and explored upon transfection into B16-F1 cells lacking endogenous WRC (CYFIP1/2 KO#3; Reference [7]). Single cell analyses revealed the complete absence of rescue of lamellipodia formation with this variant (Figure 4A,B). Immunoblotting using bulk cell extracts of CYFIP1/2 null cells transiently transfected with WT CYFIP1 or E1150Dfs*3-CYFIP1 indicated that the latter variant is only weakly expressed as compared to WT CYFIP1 (Figure 4C). The lack of lamellipodia rescue, however, cannot just be explained by reduced protein levels, as incomplete CYFIP1/2 CRISPR KO cell clones harboring only residual amounts of CYFIP proteins were still able to form lamellipodia, at least in part (Figure S3). Instead, the E1150Dfs*3 mutant was unable to incorporate into endogenous WRC, as it was unable to co-precipitate any detectable WRC subunit, as exemplified by Nap1, WAVE, and Abi-1 in wildtype B16-F1 cells. In contrast, all of aforementioned, endogenous WRC subunits were readily co-immunoprecipitated by both, EGFP-tagged WT and R87C-CYFIP1 (Figure 4D). The observed weak expression of E1150Dfs*3 variant also correlated with reduced protein half-life, as determined by treatment with the protein synthesis inhibitor cycloheximide (Figure 4E), indicating impaired protein stability as compared to WT. It is therefore tempting to speculate that the reduced half-life observed for E1150Dfs*3-CYFIP1 is due to its incapability of incorporating into WRC. The inability of E1150Dfs*3 to associate with WRC subunits then also explains its incapability of rescuing the downregulation of WAVE expression in CYFIP1/2 KOs, as opposed to WT CYFIP1 (Figure 4C). Together, all these data reveal that E1150Dfs*3 constitutes a “null” allele unable to associate with and thus stabilize remaining WRC subunits, thus failing to operate as functional unit linking Rac activation to WRC function.

## 4. Discussion

In this study, we systematically analyzed CYFIP2 mutations, reported to cause intellectual disability [23,24]. WRC is a highly conserved protein complex. We could previously show that the regulation of WRC by Rac GTPase is conserved from *D. discoideum* to mammals [7]. Furthermore, both Rac binding sites, as well as the WH2- and C-region contact sites of WAVE, are conserved in CYFIP1 and CYFIP2, strongly suggesting that their principle regulation by Rac are indistinguishable. In this study, we show that CYFIP mutations positioned within or in close proximity to the C region binding site behave similarly to constitutively active WRC. Upon abrogation of the Rac-binding A site, which is crucial for allosteric activation of WRC, CYFIP1 mutations R87C, I640M, E641K, D700H, Q701R can all rescue lamellipodia formation, in a fashion comparable to the known constitutively active mutant WCA* [7,10]. One mutation (A456P), slightly distinctly positioned within CYFIP as compared to aforementioned ones, displayed a more moderate but clearly detectable activation as well. In a complementary assay, in which cellular Rac levels were reduced in the absence of endogenous CYFIP1 and -2, the R87C mutation again behaved in a fashion comparable to WCA*, i.e., caused a significant increase of the cell fraction capable of lamellipodia formation as compared to wildtype CYFIP1. Importantly, such an increase was also observed for CYFIP2-R87C now relative to CYFIP2, confirming that CYFIP2 mutations are readily transferable into the functional context of the highly conserved CYFIP1.

In spite of the lack of detectable WRC activation in the absence of a functional A site, one particular mutation turned out to be an outstanding example of a mutant mediating WRC activation (Y108A), which was due to its unique mechanism of WRC activation. Our experiments revealed that Y108A renders WRC more active than wildtype through either directly modulating Rac binding or at least through impacting on the consequences of Rac binding to the A site, culminating in enhanced WRC activation. This observation is fully consistent with the position of Y108, which is closely located to the A site, but its precise mechanism of action will have to be determined by future, detailed biochemical and/or structural studies. Notwithstanding this, it is also worth noting that publicly available databases indicate Y108 to constitute by far the most phosphorylated residue in CYFIP proteins (www.phosphosite.org). It is reasonable to assume, therefore, that CYFIP1/2 phosphorylation at this site can fine-tune the extent of Rac-mediated WRC activation through the A site. Future efforts will aim at solidifying this hypothesis and identifying the potential kinase(s) capable of operating in this specific signaling mechanism.

Although all aforementioned mutations promoted WRC activation, albeit with clearly distinct mechanisms, one mutation causing truncation of the C-terminal 100 amino acids (E1150Dfs*3) generated a loss-of-function variant, entirely abolished for assembling into WRC and for driving lamellipodia formation. This appeared puzzling at first glance. However, WAVE1 mutations causing partial WCA domain truncations, thus behaving as dominant negatives, have also been shown to be causative for ID [21]. All this suggests that a precisely tuned, narrow window of WRC activity in the human brain is required for its proper development and function, with both hyper-activation and downregulation leading to similarly drastic consequences. Supporting this hypothesis, a recent study also suggested that tight regulation of Rac activity is required for proper neurological function, and that both hyper- and hypoactivation of Rac signaling can cause ID [28]. However, both C-terminally truncated and thus dominant-negative WAVE1 mutants and activating CYFIP2 mutants will likely display enhanced Rac binding when assembled into WRC, at least in theory. More specifically, it is well established that experimental interference with the CYFIP-WCA domain interaction (i.e., the WCA* mutation employed above, see also [7]) dramatically enhances Rac binding. This is because Rac binding-mediated WRC activation competes with the CYFIP-WCA domain interaction, and mutating the WCA domain contact sites in CYFIP and deleting WAVE’s WCA domain is thus expected to cause analogous effects with respect to Rac binding [10]. It is possible therefore that both WAVE truncation and activating CYFIP2 mutations can similarly sequester away active Rac, thereby suppressing Rac functions and its associated effector pathways. This could work in a fashion comparable to what has recently been suggested for the Rac binding protein FAM49B [29,30,31]. Such a scenario would also fit the observation that certain missense mutations in Rac1 leading to ID cause its loss of function [22], as well as the model that signaling of the “Rac1-antagonist” RhoA is typically upregulated and Rac signaling suppressed in ID [32].

It was previously suggested that WRC activation occurs via disassembly of the complex, leading to a CYFIP/Nap/Abi and a WAVE/HSPC300 subcomplex, respectively [11], as well as that ID-related CYFIP2 mutations would act by destabilizing WRC, favoring its disassembly [24]. This view, however, is challenged by numerous recent observations that established that following WRC activation, the complex stays intact [7,9,10]. In line with the latter view, we found the activating mutation R87C to normally interact with WRC components, including WAVE, while the truncating mutation E1150Dfs*3 (that fails to cause lamellipodia formation) was found to show no interaction with WRC components. All these data are in accordance with a revised model of WRC activation, in which activation occurs through release of WCA domain for Arp2/3 complex activation and actin filament branching, while the WRC unit remains intact [7,9,10].

Taken together, our results described here demonstrate that ID-causing CYFIP2 mutations can modulate WRC activity by various, distinct mechanisms, and even display opposing effects on WRC activity, in contrast to what has been anticipated previously [24]. They also emphasize the need for robust and clean experimental readouts, such as phenotypic analyses of cell lines derived from distinct, CRISPR/Cas9-based gene disruption experiments combined with specific rescue approaches.

## Supplementary Materials

The following are available online at https://www.mdpi.com/2073-4409/9/6/1355/s1, Figure S1: Sequence alignment of CYFIP paralogs, Figure S2: CYFIP1 and CYFIP2 have largely redundant functions within WRC. Figure S3: A broad range of CYFIP protein levels can mediate lamellipodia formation.
