# Supplementary material for: Molecular Dissection of Neurodevelopmental Disorder-Causing Mutations in CYFIP2
Source: Cells. 2020 May 29;9(6):1355. doi: 10.3390/cells9061355 (PMC7348743; doi:10.3390/cells9061355)
Supplement: Supplementary file 1 [file cells-09-01355-s001.pdf]

cells

MDPI

Supplementary Materials

Molecular Dissection of Neurodevelopmental Disorder-Causing Mutations in CYFIP2

Matthias Schaks [1,2], Michael Reinke [3], Walter Witke [3] and Klemens Rottner [1,2,4,*]

1 Division of Molecular Cell Biology, Zoological Institute, Technische Universität Braunschweig, 38106 Braunschweig, Germany; m.schaks@tu-braunschweig.de
2 Department of Cell Biology, Helmholtz Centre for Infection Research, 38124 Braunschweig, Germany
3 Institute of Genetics, University of Bonn, 53115 Bonn, Germany; reinke.michael@uni-bonn.de (M.R.); w.witke@uni-bonn.de (W.W.)
4 Braunschweig Integrated Centre of Systems Biology (BRICS), 38106 Braunschweig, Germany
* Correspondence: k.rottner@tu-braunschweig.de; Tel.: +49-17-1819-5199

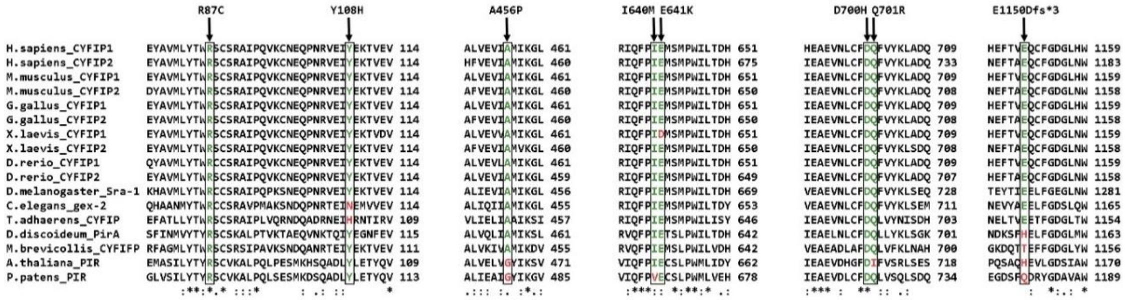

Figure S1. Sequence alignment of CYFIP paralogs. A multiple sequence alignment was performed on CYFIP paralogs. Intellectual disability-related CYFIP2 mutations are highlighted. Amino acid conservation and divergence during evolution are indicated by green and red colours, respectively. CYFIP1 mutations that have been employed in this study are indicated by arrows at the top.

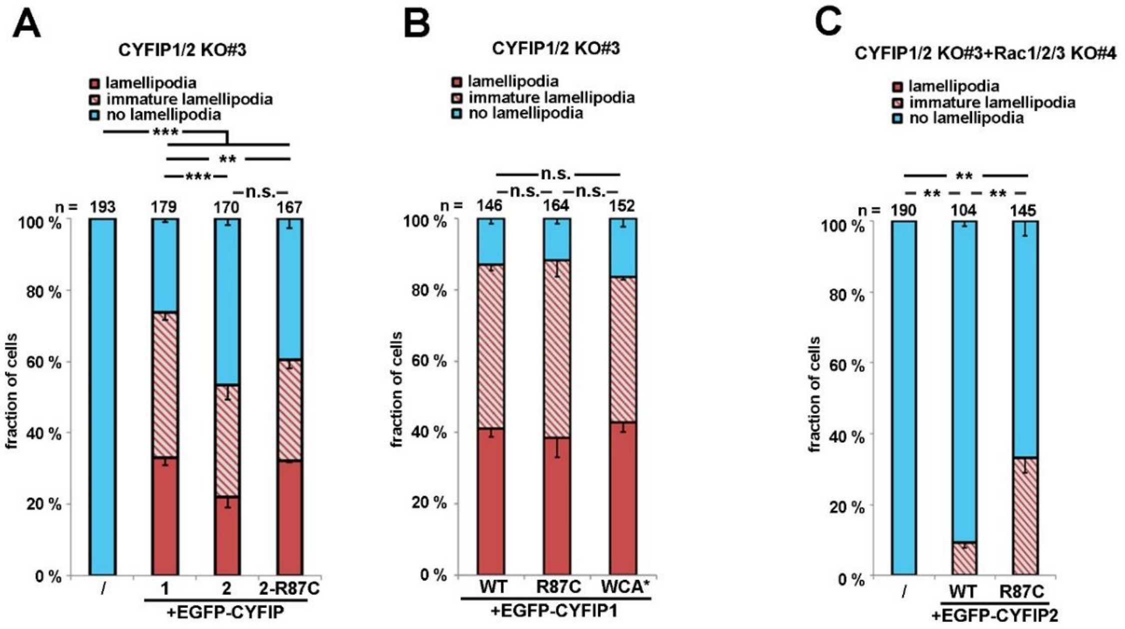

Figure S2. CYFIP1 and CYFIP2 have largely redundant functions within WRC. (**A**,**B**,**C**) Quantification of lamellipodia formation in indicated B16-F1 derived KO cells and transfected with indicated, EGFP-tagged CYFIP1 or CYFIP2 constructs, performed as described for Figure 1A. Statistical significance was assessed by two-sample, two-sided t-test. n.s.: not statistically significant; $^{**}p < 0.01$; $^{***}p < 0.001$.

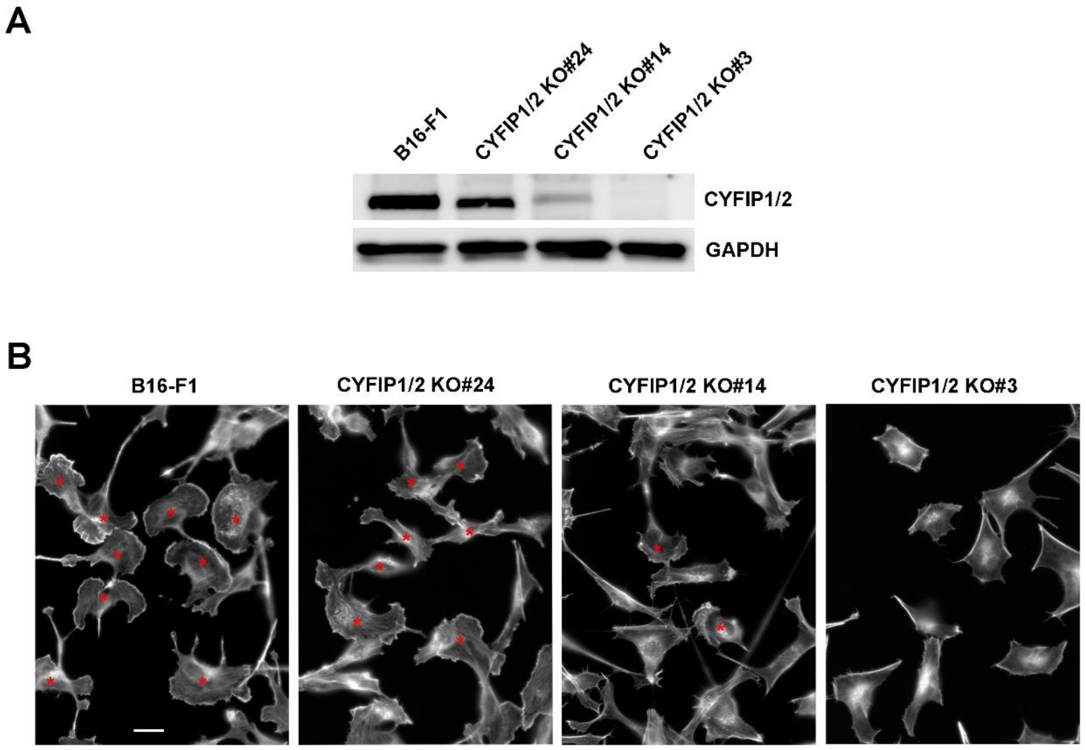

Figure S3. A broad range of CYFIP protein levels can mediate lamellipodia formation. (**A**) Western blotting of different CYFIP1/2 KO cell clones harbouring distinct amounts of CYFIP protein levels. (**B**) Fluorescence images of phalloidin stainings showing cell morphologies of indicated cell lines. Red asterisks mark cells with lamellipodia. Note that even clone #14 displaying strongly reduced CYFIP protein levels (see A) is still capable of occasional lamellipodia formation, whereas only CYFIP1/2-deficient cells (clone #3, see also right lane in A) are abolished for lamellipodia formation in these conditions. Scale bar, 20 μm.
